# Impact of Next-generation Sequencing Defined Human Immunodeficiency Virus Pretreatment Drug Resistance on Virological Outcomes in the ANRS 12249 Treatment-as-Prevention Trial

**DOI:** 10.1093/cid/ciy881

**Published:** 2018-10-15

**Authors:** Anne Derache, Collins C Iwuji, Kathy Baisley, Siva Danaviah, Anne-Geneviève Marcelin, Vincent Calvez, Tulio de Oliveira, François Dabis, Kholoud Porter, Deenan Pillay

**Affiliations:** 1 Africa Health Research Institute, Mtubatuba, South Africa; 2 Sorbonne University, l’université Pierre et Marie Curie, Institut national de la santé et de la recherche médicale, Institut Pierre Louis d’Epidémiologie et de Santé Publique Unité Mixte de Recherche en Santé (IPLESP UMRS 1136), Paris, France; 3 Department of Global Health and Infection, Brighton and Sussex Medical School; 4 Institute for Global Health, University College London, United Kingdom; 5 KwaZulu-Natal Research Innovation and Sequencing Platform, School of Laboratory Medicine and Medical Sciences, College of Health Sciences, University of KwaZulu-Natal, Durban, South Africa; 6 Université de Bordeaux, Institut de Santé Publique d’Epidémiologie et de Développement, Centre Institut national de la santé et de la recherche médicale 1219, France; 7 Division of Infection and Immunity, University College London, United Kingdom

**Keywords:** HIV, pretreatment drug resistance, antiretroviral therapy, next-generation sequencing, virological response

## Abstract

**Background:**

Previous studies in human immunodeficiency virus (HIV)-positive individuals on thymidine analogue backbone antiretroviral therapy (ART) with either nevirapine or efavirenz have suggested poorer virological outcomes in the presence of pretreatment drug resistance (PDR). We assessed the impact of PDR on virological suppression (VS; <50 copies/mL) in individuals prescribed primarily tenofovir/emtricitabine/efavirenz in rural KwaZulu-Natal within a treatment-as-prevention trial.

**Methods:**

Among 1557 HIV-positive individuals who reported no prior ART at study entry and provided plasma samples, 1328 individuals with entry viral load (VL) >1000 copies/mL had next-generation sequencing (NGS) of the HIV *pol* gene with MiSeq technology. Results were obtained for 1148 individuals, and the presence of PDR was assessed at 5% and 20% detection thresholds. Virological outcome was assessed using Cox regression in 837 of 920 ART initiators with at least 1 follow-up VL after ART initiation.

**Results:**

PDR prevalence was 9.5% (109/1148) and 12.8% (147/1148) at 20% and 5% thresholds, respectively. After a median of 1.36 years (interquartile range, 0.91–2.13), mostly on fixed-dose combination tenofovir/emtricitabine/efavirenz, presence of both nonnucleoside reverse transcriptase inhibitor (NNRTI)/nucleoside reverse transcriptase inhibitor PDR vs no PDR was associated with longer time to VS (adjusted hazard ratio [aHR], 0.32; 95% confidence interval [CI], 0.12–0.86), while there was no difference between those with only NNRTI PDR vs no PDR (aHR, 1.05; 95% CI, 0.82–1.34) at the 5% threshold. Similar differences were observed for mutations detected at the 20% threshold, although without statistical significance.

**Conclusions:**

NGS uncovered a high prevalence of PDR among participants enrolled in trial clinics in rural KwaZulu-Natal. Dual-class PDR to a mainly tenofovir/emtricitabine/efavirenz regimen was associated with poorer VS. However, there was no impact of NNRTI PDR alone.

**Clinical *T*rials *T*egistration:**

NCT01509508; South African National Clinical Trials Register: DOH-27-0512-3974.

## Teaser

We documented a high prevalence of pretreatment drug resistance (PDR) among participants enrolled in trial clinics in rural KwaZulu-Natal. Dual-class PDR to a first-line tenofovir/emtricitabine/efavirenz regimen was associated with poorer virological suppression. However, there was no impact of nonnucleoside reverse transcriptase inhibitor PDR alone.

**(See the Editorial Commentary by Shafer and Frenkel on pages 215–7.)**

Human immunodeficiency virus (HIV) antiretroviral therapy (ART) scale-up in eastern and southern Africa has been a great success, with a doubling of the number of people on ART since 2010, reaching 10.3 million people in 2016, and with a 36% decline in the number of AIDS-related deaths [1]. Despite the benefits of ART for individuals and populations [2, 3], expanding ART access and longer time on therapy might increase emergence and transmission of drug resistance (DR) [4], which could potentially compromise public ART programs in settings that use standardized first-line regimens. The majority of studies in sub-Saharan Africa (Supplementary Table 1) have shown a detrimental impact of pretreatment DR (PDR) on virological outcomes in individuals prescribed first-line ART mainly comprising a thymidine analogue backbone (zidovudine [ZDV] or stavudine [d4T] combined with either efavirenz [EFV] or nevirapine [NVP]) [4–9]. Four of these studies accounted for ART adherence [4–6, 8]. Fewer, generally smaller studies, that evaluated populations prescribed mainly older first-line ART regime, have not shown a similar association [10–13].

Within the Treatment-as-Prevention (TasP) trial, a cluster-randomized trial undertaken in an HIV hyperepidemic setting in rural KwaZulu-Natal, South Africa [14], we estimated the prevalence of PDR using next-generation sequencing (NGS) technologies among HIV-positive participants who reported not to be on ART at entry into trial clinics. We evaluated the association between PDR and the response to first-line ART (predominantly fixed-dose combination [FDC] tenofovir/emtricitabine/efavirenz [TDF/FTC/EFV; Atripla]) in individuals who initiated ART within the trial.

## METHODS

### Ethics Statement

The Biomedical Research Ethics Committee (BFC 104/11) at the University of KwaZulu-Natal and the Medicines Control Council of South Africa approved the trial. All trial participants gave written or witnessed thumbprint informed consent prior to undertaking any study procedures.

### Study Design and Trial Setting

The French National Agency for Aids and Viral Hepatitis Research (ANRS) 12249 TasP trial was implemented in the Hlabisa subdistrict in rural KwaZulu-Natal [14], one of the poorest communities in South Africa, with a high unemployment rate [15]. This was a cluster-randomized trial undertaken between March 2012 and June 2016 in 22 clusters (2 × 11) [16, 17]. Participants residing in the intervention clusters were offered ART after HIV diagnosis, regardless of their CD4 count, whereas participants in control clusters were offered ART according to the prevailing South African guidelines.

### Study Procedures and Laboratory Methods

Individuals aged ≥16 years who tested positive for HIV through home-based rapid test or who self-reported to be HIV positive were referred to the trial clinics in their cluster, regardless of their ART status.

Individuals who linked to care were asked to complete study questionnaires and provide plasma samples at their first trial clinic visit, then at 3 months, 6 months, and every 6 months thereafter if they initiated ART. Plasma samples were used for viral load (VL) testing, using the Abbott RealTime HIV-1 m2000rt (Abbott Molecular Inc., Des Plaines, IL), as well as for DR testing in the Africa Health Research Institute diagnostic laboratory. Individuals visited the clinics monthly for their ART prescription, where adherence was measured using the visual analogue scale (VAS) [18]. Participants were asked to mark their level of adherence in the previous 4 days on a VAS that ranged from 0 (no ART tablets taken) to 100% (all ART tablets taken). Adherence was suboptimal if ≤95%.

Plasma samples with VL ≥1000 copies/mL were characterized for HIV *pol* with NGS, using MiSeq technology, according to an adapted protocol from Gall et al (Supplementary Methods 1 and Supplementary Table 2) [19]. After reads assemblies using Geneious 10.0.6 software [20] and quality control of NGS data, DR mutations (DRMs) were called at a threshold of 5% (Supplementary Methods 2). Resistant variants were included in the analysis when they were also detected by another application available in BaseSpace MiCall [21]. The DRMs were documented using the World Health Organization (WHO) 2009 surveillance of DRM [22]. PDR prevalence and impact were estimated from DRMs detected at a >5% confidence level of real mutation detection and a >20% level of detection reached by Sanger population sequencing, the most common technique used in DR testing.

### Statistical Analyses

The characteristics of individuals who had NGS sequence data at baseline with and without PDR were tabulated. Characteristics of individuals who initiated ART in the trial, had NGS sequence data at baseline, and had at least 1 follow-up VL measurement (ie, so included in the analysis of VS) were tabulated and compared with those individuals who were missing VL at follow-up. We checked for completeness of VL measurements in those with and without PDR during the first 12 months after ART initiation to exclude ascertainment bias.

Categorical variables were summarized using frequencies and proportions and compared using χ^2^ tests. Continuous variables were summarized using median and interquartile ranges (IQRs) and compared using Mann-Whitney tests.

We computed the overall proportions of individuals with any PDR and nonnucleoside reverse transcriptase inhibitor (NNRTI) at 5% and 20% detection thresholds. We examined the association between PDR stratified based on predicted response to the antiretroviral drugs prescribed (no PDR, only NNRTI PDR, or both nucleoside reverse transcriptase inhibitor [NRTI]/NNRTI PDR) and time to VS. Two separate analyses were undertaken for time to VS; PDR was defined as whether or not mutations were present at the 20% threshold and then at the 5% threshold. Kaplan-Meier methods were used to estimate time to VS in the 3 PDR categories, which were compared using the log-rank test. Individuals entered the analysis at the date of ART initiation; those who did not achieve VS were censored at the date of their last VL measurement. Cox regression was used to estimate hazard ratios (HRs) and 95% confidence intervals (CIs) for the association of PDR and other factors with VS. Factors that were associated with VS at *P* < .15 in the unadjusted analysis were included in a multivariable model. Age and sex were retained a priori as potential confounders. CD4 count and age were included in the model as continuous covariates. In order to allow for a nonlinear relationship between CD4 count, age, and time to VS, we used fractional polynomial functions, which provide a flexible way to model the shape of the relationship of a continuous variable with the outcome [23]. We used a set of defined powers (–2, –1, –0.5, 0.5, 1, 2, and ln(x)) and a maximum of 2 power terms in the model. The differences in model deviances were compared. The linear model was used if the improvement in fit was not statistically significant at *P* < .05. Mean VAS adherence during follow-up was calculated by taking the average adherence in the visits prior to achieving VS in those who achieved VS or the average adherence in the visits prior to censoring in those who did not achieve VS. Missing adherence measurements were omitted. VAS adherence was transformed into a categorical variable using clinically meaningful cutoffs. VL was handled in a similar manner.

After fitting the full model, the proportional hazard assumption was tested both globally and for individual covariates by regressing the scaled Schoenfeld residuals on time. The null hypothesis was that the slope was zero, that is, that the log HR function was constant over time.

## RESULTS

### Cohort Description

**Figure 1.**
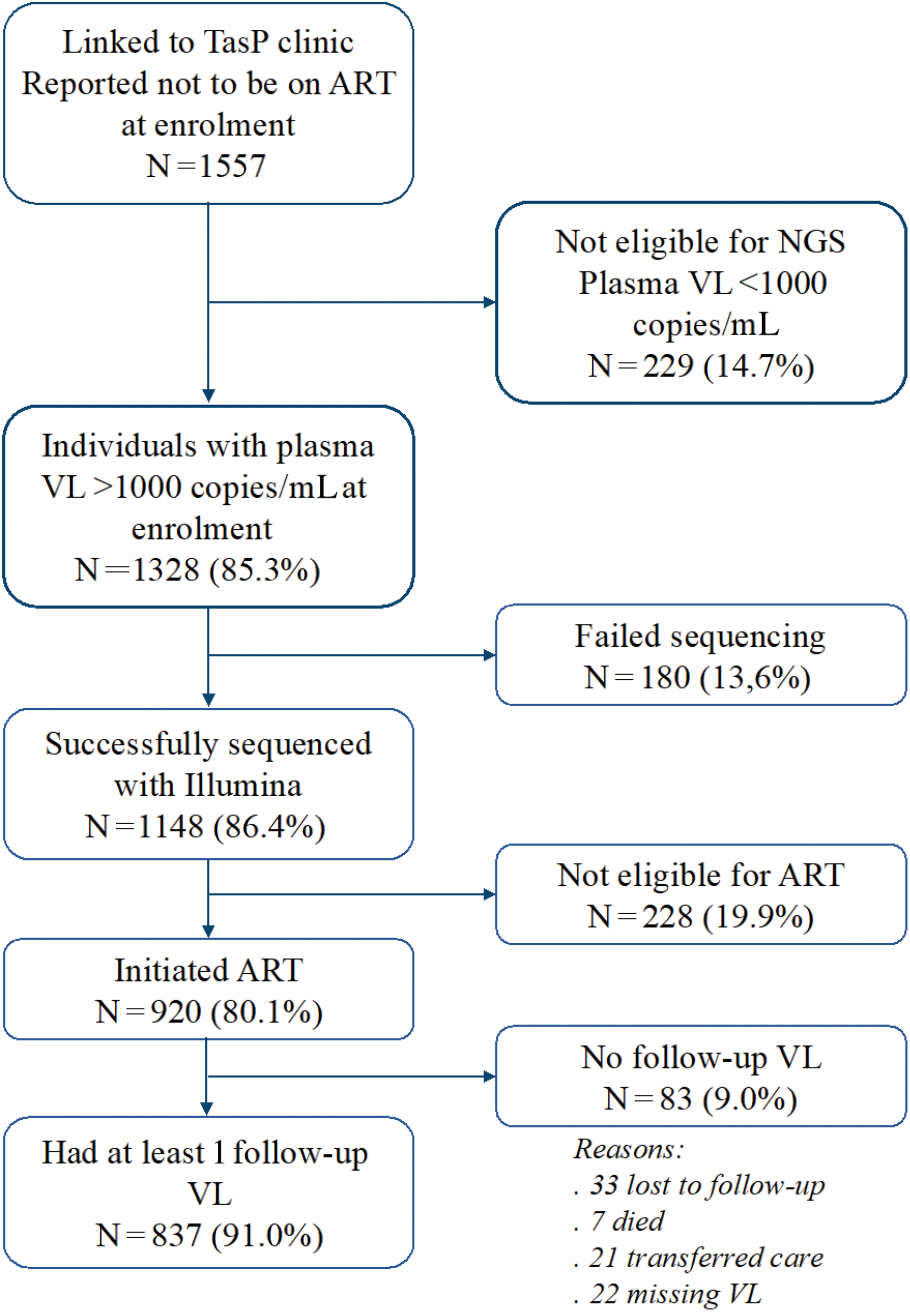
Cohort flow chart. Abbreviations: ART, antiretroviral therapy; NGS, next-generation sequencing; TasP, Treatment-as-Prevention; VL, viral load.

Of the 1557 participants who reported not to be on ART at entry, 1328 (85.3%) had a VL >1000 copies/mL, of whom 1148 (86.4%) had successful NGS of the HIV *pol* gene (consensus sequences available in GenBank, accession numbers MH709380–MH710527). Of the 1148 with NGS data, 920 (80.1%) initiated ART within the trial, of whom 837 individuals had at least 1 VL result after ART initiation (Figure 1).

### Prevalence of Any PDR or NNRTI DRM

**Figure 2.**
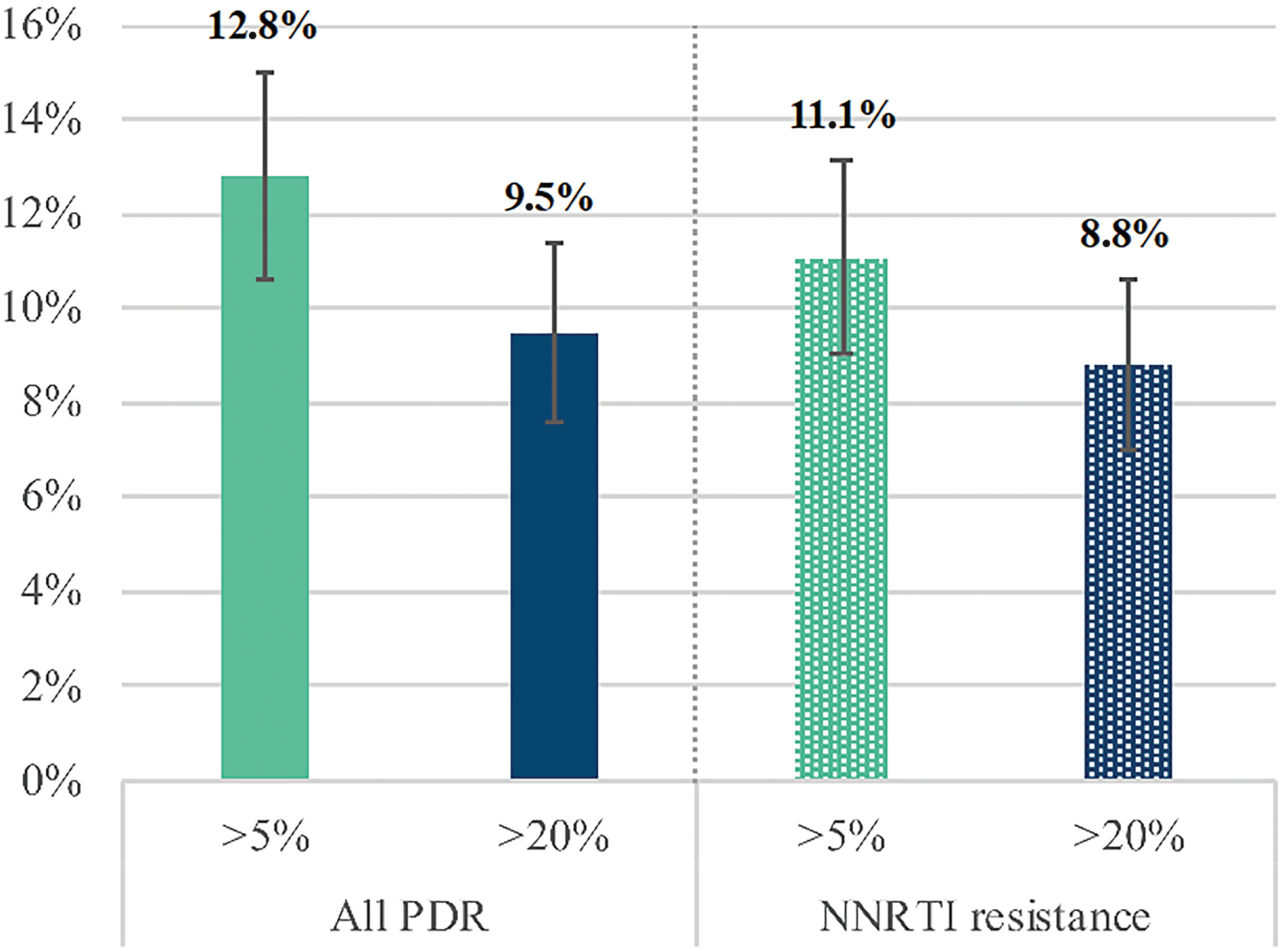
Prevalence of any pretreatment drug resistance and nonnucleoside reverse transcriptase inhibitor resistance among 1148 participants with next-generation sequencing data detected at 5% and 20% detection thresholds. Abbreviations: NNRTI, nonnucleoside reverse transcriptase inhibitor; PDR, pretreatment drug resistance.

Of the 1148 participants who had their virus successfully sequenced, 109 (9.5%) had at least 1 PDR mutation detected at 20% threshold, NNRTI resistance being predominant with a prevalence of 101/1148 (8.8%). The number of participants with any PDR mutation increased to 147 (12.8%) when minority variants were accounted for at 5% threshold (Figure 2). Prevalence of NRTI resistance was low, with 12 (1.1%) and 23 (2.0%) participants out of 1148 having NRTI DRM detected at 20% and 5% thresholds, while protease inhibitor resistance was found in 8 (0.7%) and 16 (1.4%) individuals, respectively. Detailed descriptions of the DRM are presented in Supplementary Figures 1 and 2. Among those with resistance, dual-class NRTI/NNRTI DRMs were found in 6/109 (5.5%) and 11/147 (7.8%) participants with PDR at 20% and 5% thresholds, respectively (Supplementary Table 3).

The median age of the majority of participants with virus sequences was 32.9 years (IQR, 25.6–45.2), with characteristics described in Table 1. The median CD4 count at clinic presentation was 405 cells/mm^3^ (IQR, 261–559), and the median VL was 4.5 log10 copies/mL (IQR, 3.9–5.2). There was no difference in the median age of individuals with sequences (n = 1148) and those without (n = 409; 32.9 years [IQR, 25.6–45.2] vs 33.5 years [IQR, 26.6–45.6]; *P* = .67). A higher proportion of females than males had no virus sequences (28.1% vs 21.4%; *P* = .008).

**Table 1.** Demographic and Clinical Characteristics of All Participants Assessed for Pretreatment Drug Resistance^a^

Characteristics of Individuals With Sequences	Total N = 1148 (%)	Individuals Without Pretreatment HIV Drug Resistance n = 1039 (%)	Individuals With Pretreatment HIV Drug Resistance n = 109 (%)
Age (y)			
Median age (IQR)	32.9 (25.6–45.2)	33.3 (25.8–45.8)	30.0 (25.0–36.4)
16–29	463 (40.3)	409 (39.4)	54 (49.5)
30–39	298 (26.0)	267 (25.7)	31 (28.4)
40–49	178 (15.5)	168 (16.2)	10 (9.2)
>50	202 (17.6)	189 (18.2)	13 (11.9)
Missing	7 (0.6)	6 (0.6)	1 (0.9)
Sex			
Female	807 (70.3)	729 (70.2)	78 (71.6)
Male	341 (29.7)	310 (29.8)	31 (28.4)
CD4 at presentation			
Median (IQR) (cells/mm^3^)	404 (261–559)	405 (261–559)	383 (263–533)
<350	448 (39.0)	404 (38.9)	44 (40.4)
350–500	299 (26.1)	270 (26.0)	29 (26.6)
>500	379 (33.0)	348 (33.5)	31 (28.4)
Missing	22 (1.9)	17 (1.6)	5 (4.6)
Viral load (copies/mL)			
Median (log10)	4.5 (3.9–5.2)	4.5 (3.9–5.2)	4.6 (4.1–5.1)
<10000	309 (26.9)	285 (27.4)	24 (22.0)
10000–100000	478 (41.6)	429 (41.3)	49 (45.0)
>100000	356 (31.0)	320 (30.8)	36 (33.0)
Missing	5 (0.4)	5 (0.5)	0 (0.0)
Education			
Primary or less	483 (42.1)	432 (41.6)	51 (42.5)
Some secondary	427 (37.2)	385 (37.1)	47 (39.2)
Secondary or higher	234 (20.4)	218 (21.0)	22 (18.3)
Missing	4 (0.4)	4 (0.3)	0 (0.0)
Marital status			
Never married	1009 (87.9)	904 (87.0)	105 (96.3)
Married	92 (8.0)	89 (8.6)	3 (2.8)
Divorced/separated	43 (3.8)	42 (4.0)	1 (0.9)
Missing	4 (0.4)	4 (0.4)	0 (0.0)
Employment			
Employed	166 (14.5)	155 (14.9)	11 (10.1)
Student	60 (5.2)	53 (5.1)	7 (6.4)
Unemployed	917 (79.9)	826 (79.5)	91 (83.5)
Missing	5 (0.4)	5 (0.5)	0 (0.0)
Receiving government grants			
Yes	662 (57.7)	597 (57.5)	65 (59.6)
No	473 (41.2)	429 (41.3)	44 (40.4)
Missing	13 (1.1)	13 (1.3)	0 (0.0)

Abbreviations: HIV, human immunodeficiency virus; IQR, interquartile range.

^a^Pretreatment drug resistance is defined by next-generation sequencing only.

### Association of Pretreatment Drug Resistance With Virologic Suppression

Of the 920 individuals who initiated ART (96.3% started Atripla) and had virus sequence data, 837 had at least 1 follow-up VL and were used to examine the impact of PDR on response to therapy. There was no statistically significant difference in the completeness of VL measurements at each visit between individuals with and without PDR during the first 12 months of ART (Supplementary Table 4). The median age was 34.3 years, 72% were female, and 83.5% had an overall mean VAS adherence ≥95% (Table 2). The 83 participants without VL data were younger than those with VL data (median age, 29.5 years [IQR, 23.5–41.6] vs 34.3 years [IQR, 27.3–46.5]; *P* = .02) and a higher proportion were male (42% vs 28%; *P* = .009). The prevalence of any PDR at the 20% threshold in participants with and without VL data (9.4% vs 12.1%; *P* = .44, respectively) was similar to that in all individuals with sequences (9.5%).

**Table 2.** Baseline Characteristics of Individuals Contributing to the Analysis of Virological Suppression

Characteristic	In Analysis n = 837 (%)	Missing Viral Load N = 83 (%)	*P* Value
Age at initiation (y)			
Median age (IQR)	34.3 (27.3, 46.5)	29.5 (23.5, 41.6)	.02
16–29	290 (34.6)	43 (51.8)	…
30–39	246 (29.4)	15 (18.1)	…
40–49	133 (15.9)	9 (10.8)	…
>50	166 (19.8)	13 (15.7)	…
Missing	2 (0.2)	3 (3.6)	…
Sex			
Female	599 (71.6)	48 (57.8)	.009
Male	238 (28.4)	35 (42.2)	…
CD4 at initiation			
Median (IQR) (cells/mm^3^)	348 (227, 480)	399 (235, 521)	.630
≤350	418 (49.9)	37 (44.6)	…
350–500	230 (27.5)	20 (24.1)	…
>500	182 (21.7)	22 (26.5)	…
Missing	7 (0.8)	4 (4.8)	…
Viral load (copies/mL)			
Median (log copies/mL)	4.6 (4.0, 5.2)	4.6 (3.9, 5.2)	.818
<10000	200 (23.9)	22 (26.5)	…
10000–100000	350 (41.8)	36 (43.3)	…
>100000	285 (34.1)	25 (30.1)	…
Missing	2 (0.2)	0 (0.0)	…
Adherence (%)			
<95	126 (15.1)	…	…
≥95	699 (83.5)	…	…
Missing	12 (1.4)	…	…
Antiretroviral therapy regimen			.001
TDF+FTC+EFV	806 (96.3)	73 (88.0)	…
TDF+3TC+EFV	6 (0.7)	2 (2.4)	…
AZT+3TC+EFV	18 (2.2)	3 (3.6)	…
D4T+3TC+EFV	1 (0.1)	…	…
AZT+3TC+PI	1 (0.1)	…	…
Missing	5 (0.6)	5 (6.0)	…

Abbreviations: 3TC, lamivudine; AZT, zidovudine; D4T, stavudine; EFV, efavirenz; FTC, emtricitabine; IQR, interquartile range; TDF, tenofovir.

**Figure 3.**
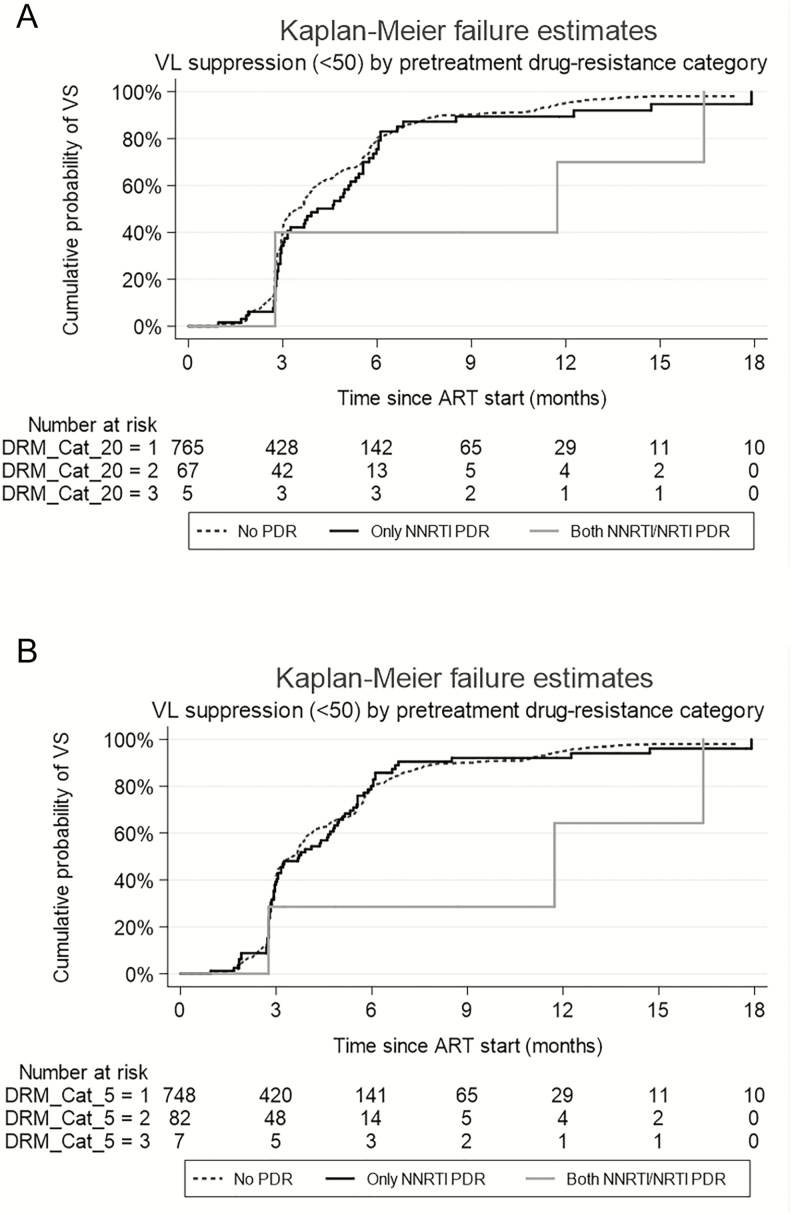
Kaplan-Meier plot of the cumulative probability of virological suppression since antiretroviral therapy start; stratified by class of pretreatment drug resistance at the 20% (A) and 5% (B) detection thresholds. Abbreviations: ART, antiretroviral therapy; DRM, drug-resistance mutation; NRTI, nucleoside reverse transcriptase inhibitor; NNRTI, nonnucleoside reverse transcriptase inhibitor; PDR, pretreatment drug resistance; VL, viral load; VS, virological suppression.

Among the 837 HIV-positive individuals who contributed to the analysis, 748 had no PDR, 82 had NNRTI PDR only, and 7 had both NRTI and NNRTI PDR at the 5% threshold. At the 20% threshold, the corresponding numbers were 765, 67, and 5, respectively. Participants were followed for a median of 1.36 years (IQR, 0.91–2.13) after ART initiation. At the 20% detection threshold, time to VS was longer for those with both NRTI/NNRTI PDR than those without any PDR (median, 11.73 months [IQR, 2.76–16.39] vs 3.45 months [IQR, 2.79–5.75]), while there was no significant difference between those with only NNRTI PDR compared to those with no PDR (median, 4.11 months [IQR, 2.86–5.98] vs 3.45 months [IQR, 2.79–5.75]; Figure 3A) (log-rank test overall; *P* = .10). At the 5% detection threshold, time to VS was longer for those with both NRTI/NNRTI PDR than those without any PDR (median, 11.73 months [IQR, 2.76–16.39] vs 3.48 months [IQR, 2.79–5.78]), while there was no difference between those with only NNRTI PDR compared to those with no PDR (median, 3.71 months [IQR, 2.79–5.55] vs 3.48 months [IQR, 2.79–5.78]; Figure 3B) (log-rank test overall; *P* = .09). The median time to achieve VS, overall, was 3.61 months (IQR, 2.79–5.78). The overall cumulative probability of VS at 12 months was 94.5% (95% CI, 92.7–96.0).

In unadjusted Cox models, for resistant variants detected at 20% (Table 3), there was an association between presence of both NRTI/NNRTI PDR with longer time to VS, but this did not reach statistical significance (HR, 0.42; 95% CI, 0.16–1.12). However, there was no association with VS for those with only NNRTI PDR (HR, 0.84; 95% CI, 0.64–1.11). Factors associated with longer time to VS were being male and having a high VL at baseline (>100000 copies/mL), while a mean VAS adherence of ≥95% and a higher CD4 count at initiation were associated with shorter time to VS. In a multivariable Cox regression model that adjusted for age, sex, CD4 count, and VL at ART initiation and adherence, the association between having both NRTI/NNRTI PDR and VS remained virtually unchanged from the unadjusted model (adjusted (a)HR, 0.41; 95% CI, 0.15–1.10), with attenuation of the effect of association between having only NNRTI PDR and VS (aHR, 0.90; 95% CI, 0.68–1.18). Having a high baseline VL was independently associated with significantly longer time to VS, while VAS adherence ≥95% remained independently associated with shorter time to VS.

**Table 3.** Factors Associated With Virologic Suppression in Adults With Pretreatment Drug Resistance Detected at the 20% Threshold

Characteristic	Unadjusted HR (95% CI)	*P* Value	Adjusted HR (95% CI)	*P* Value
Pretreatment drug resistance	…	.06	…	.09
No PDR	1	…	1	…
Only NNRTI PDR	0.84 (0.64–1.11)	…	0.90 (0.68–1.18)	…
Both NNRTI/NRTI PDR	0.42 (0.16–1.12)	…	0.41 (0.15–1.10)	…
Age at initiation/5 years	1.02 (1.00–1.05)	.11	1.03 (1.00–1.06)	.06
Sex	…	.01	…	.69
Female	1	…	1	…
Male	0.82 (0.70–0.96)	…	0.97 (0.82–1.14)	…
CD4 at initiation (100 cells/mm^3^)	1.06 (1.03–1.09)	<.001	1.03 (1.00–1.06)	.10
Viral load (copies/mL)	…	<.001	…	<.001
≤10000	1	…	1	…
10000–100000	0.74 (0.61–0.88)	…	0.75 (0.62–0.90)	…
>100000	0.47 (0.38–0.56)	…	0.48 (0.39–0.59)	…
Visual analogue scale adherence (%)	…	.001	…	.003
<95	1	…	1	…
≥95	1.40 (1.14–1.73)	…	1.37 (1.11–1.70)	…

Abbreviations: CI, confidence interval; HR, hazard ratio; NNRTI, nonnucleoside reverse transcriptase inhibitor; NRTI, nucleoside reverse transcriptase inhibitor; PDR, pretreatment drug resistance.

When we repeated the analysis to take into account the presence of resistant variants detected at the 5% threshold (Table 4), we found a statistically significant association between having both NRTI/NNRTI PDR and longer time to VS (both NRTI/NNRTI PDR vs no PDR; aHR, 0.32; 95% CI, 0.12–0.86). There was no difference in time to VS between having only NNRTI PDR and no PDR (aHR, 1.05; 95% CI, 0.82–1.34).

**Table 4.** Factors Associated With Virologic Suppression in Adults With Pretreatment Drug Resistance Detected at the 5% Threshold

Characteristic	Unadjusted HR (95% CI)	*P* Value	Adjusted HR (95% CI)	*P* Value
Pretreatment drug resistance	…	.05	…	.02
No PDR	1	…	1	…
Only NNRTI PDR	0.99 (0.77-1.25)	…	1.05 (0.82–1.34)	…
Both NNRTI/NRTI PDR	0.36 (0.13-0.96)	…	0.32 (0.12–0.86)	…
Age at initiation/5 years	1.02 (1.00–1.05)	.11	1.03 (1.00–1.06)	.05
Sex	…	.01	…	.70
Female	1	…	1	…
Male	0.82 (0.70–0.96)	…	0.97 (0.82–1.14)	…
CD4 at initiation (100 cells/mm^3^)	1.06 (1.03–1.09)	<.001	1.03 (1.00–1.06)	.09
Viral load (copies/mL)	…	<.001	…	<.001
≤10000	1	…	1	…
10000–100000	0.74 (0.61–0.88)	…	0.74 (0.61–0.89)	…
>100000	0.47 (0.38–0.56)	…	0.47 (0.39–0.58)	…
Visual analogue scale adherence (%)	…	.001	…	.003
<95	1	…	1	…
≥95	1.41 (1.14–1.73)	…	1.38 (1.11–1.70)	…

Abbreviations: CI, confidence interval; HR, hazard ratio; NRTI, nucleoside reverse transcriptase inhibitor; NNRTI, nonnucleoside reverse transcriptase inhibitor; PDR, pretreatment drug resistance.

## DISCUSSION

We report the first study from the sub-Saharan HIV epidemic that explored NGS-defined DR and response to currently recommended first-line FDC therapy. The prevalence of any PDR was 9.5% at the 20% detection level and up to 13% with a detection limit of 5% among HIV-positive individuals who reported no prior ART at entry into the trial. Virological response was similar between individuals who had only NNRTI PDR and those who had no PDR. However, VS was poorer in individuals who had dual-class NRTI/NNRTI PDR than in those without PDR at the 5% threshold. The association at the 20% threshold did not reach statistical significance, most likely due to very small numbers of individuals with dual-class PDR.

Our findings contrast with those from 2 large cohort studies that addressed a similar question in sub-Saharan Africa, in which PDR defined by population sequencing was associated with virological failure or treatment switch when at least 1 drug was compromised in participants initiating first-line ART [4, 5]. The majority of participants in the cited studies were on AZT or d4T backbone in combination with either NVP or EFV. By contrast, only a third of the participants in those 2 studies were on TDF with either 3TC or FTC combined with NVP or EFV. Other similar studies in individuals prescribed predominantly older ART regimens have also shown an association between poorer virological response and PDR when at least 1 drug was compromised [6–8]. In our study with NGS-defined PDR, nearly all participants were on fixed-dose combination TDF/FTC/EFV, with VS being compromised only when PDR to at least 2 of the prescribed drugs was present. There was no difference in VS between patients with only NNRTI PDR and those with no PDR. This finding was collaborated by a descriptive study that showed that virological response was similar in individuals with only NNRTI PDR and those with no PDR if on EFV-based ART, with poorer response observed only when both NRTI and NNRTI PDR were present [9].

Our findings suggest that the combination of TDF/FTC in the presence of good adherence is potent enough to achieve short-term VS despite the presence of NNRTI PDR. TDF/FTC/EFV was found to be either equivalent or superior to its comparator arms in a study that compared 4 WHO-recommended regimens [24]. This observation was attributed to higher potency of EFV compared to NVP and the longer intracellular half of FTC-triphosphate [25] than 3TC-triphosphate [26], which could mean better forgiveness of FTC-containing regimens with missed ART doses. These factors may explain our finding of little impact of only NNRTI PDR. Some studies with small sample size have shown no association between PDR and virological outcomes [10–13].

Our PDR prevalence figures are similar to those from a recent study performed across all the South African provinces [27]. The high proportion of NNRTI resistance in that survey likely reflects the exposure of the population to NNRTI-based ART following the rollout of the national HIV treatment programs. However, NRTI mutations such as M184V, which was present in our study, were unlikely to have been transmitted because of their fitness cost to the virus. Therefore, the presence of dual-class NRTI/NNRTI mutations in our study may suggest previous ART exposure in patients who did not report it, as suggested in previous studies [27, 28]. Moreover, the use of NGS to detect minority variants at ART initiation could be clinically relevant, as poorer VS was observed in participants with NRTI/NNRTI detected at the 5% threshold.

Our study has a few limitations. About 15% of participants had VL <1000 copies/mL at entry and therefore did not have virus sequenced. If this was due to undisclosed prior ART, we could have underestimated the prevalence of PDR in the population of HIV-positive individuals who initiated or reinitiated ART. More females did not have sequences either because of low plasma VL or failure of sequencing. However, as there was no difference in the prevalence of PDR between males and females among those sequenced, we do not believe this would have biased our estimates of PDR. A small proportion (9.0%) of individuals with missing follow-up VL could not be evaluated for virological response. These individuals were younger and more likely to be male, characteristics associated with poorer VS in our cohort [29]; hence, we could have overestimated virological response in the studied sample. However, this is unlikely due to the small number of participants with missing VL.

WHO recently lowered the NNRTI DR threshold for considering a change in the first-line ART in a public health approach in low- and middle-income countries from 15% to 10% [30, 31], with dolutegravir (DTG)-based first-line ART poised to replace EFV [32, 33] because of its higher VS rates, shorter time to VS, and fewer side-effects [34, 35]. The precise impact of NRTI PDR on response to tenofovir/lamivudine/dolutegravir remains to be seen, although NNRTI PDR alone will not compromise this regimen. Moreover, there are also limited data on the use of DTG in patients with tuberculosis [36], which is prevalent in sub-Saharan Africa and in pregnancy [37]. Recent data from Botswana suggest a higher frequency of neural tube birth defects in women who conceived on DTG [38]. Hence, there would still be HIV-positive individuals for whom an EFV-based ART may be more appropriate.

In conclusion, in the setting of a community trial that involved a large study population that initiated a FDC of TDF/FTC/EFV in HIV-positive individuals, we found no association between the presence of only NNRTI PDR and VS; however, PDR to both NRTI and NNRTI was associated with longer time to VS. Good ART adherence and the high potency of TDF/FTC/EFV may have compensated for the presence of only NNRTI PDR. Studies with longer duration of follow-up in real-life public ART programs are warranted to properly quantify the effect of PDR on clinical outcomes in the African setting as new first-line regimens are rolled out.

## Supplementary Data

Supplementary materials are available at *Clinical Infectious Diseases* online. Consisting of data provided by the authors to benefit the reader, the posted materials are not copyedited and are the sole responsibility of the authors, so questions or comments should be addressed to the corresponding author.

ciy881_suppl_Supplementary_Appendix

## Notes

***Author contributions.*** C. I., A. D., D. P., and F. D. designed and implemented the study. A. D. generated and analyzed the sequencing data. C. I., A. D., and K. B. did the statistical analyses. C. I., A. D., and D. P. wrote the initial draft of the manuscript. All authors contributed to the interpretation and presentation of the findings. All authors approved the final version of the manuscript for submission.

***Acknowledgments.*** The authors thank Dr Jennifer Giandhari, Shyamala Padayachi, Zizile Sikhosana, and Sureshnee Pillay for their assistance with sample processing and sequencing. Special thanks to Prof Jean-François Delfraissy, director of American Naval Records Society. The authors thank the study volunteers for allowing us into their homes and participating in this trial and the Department of Health of South Africa for their support of this study.

***Disclaimer.*** The content is solely the responsibility of the authors and does not represent the official views of 3ie or the Bill & Melinda Gates Foundation. The funders had no role in the design, analysis, and interpretation of the study or the decision to submit for publication.

***Financial support.*** The Treatment as Prevention trial was supported by ANRS (grant ANRS 2011–375); the Deutsche Gesellschaft für Internationale Zusammenarbeit (grant 81151938); and the International Initiative for Impact Evaluation, Inc. (3ie), with support from the Bill & Melinda Gates Foundation. The authors acknowledge the Bill & Melinda Gates Foundation for also supporting the PANGEA-HIV consortium (D. P.). C. C. I. also received additional funding from the People Programme (Marie Curie Actions) (FP7/2007–2013; grant 612216). T. d. O.’s research is funded by the South African Medical Research Council (grant MRC-RFA-UFSP-01-2013/UKZN HIVEPI) and the Royal Society Newton Advanced Fellowship. The trial is conducted with the support of Merck & Co. Inc. and Gilead Sciences, which provided the Atripla drug supply. The Africa Health Research Institute receives core funding from the Wellcome Trust, which provides the platform for the population- and clinic-based research at the center.

***Potential conflicts of interests.*** C. I. received honoraria for consulting services from Gilead Sciences. A.-G. M. reports grants and personal fees from VIIV Healthcare, Gilead, MSD, and Janssen outside the submitted work. The remaining authors: No reported conflicts. All authors have submitted the ICMJE Form for Disclosure of Potential Conflicts of Interest. Conflicts that the editors consider relevant to the content of the manuscript have been disclosed.
