# Supplementary material for: Impact of Next-generation Sequencing Defined Human Immunodeficiency Virus Pretreatment Drug Resistance on Virological Outcomes in the ANRS 12249 Treatment-as-Prevention Trial
Source: Clin Infect Dis. 2018 Oct 15;69(2):207–14. doi: 10.1093/cid/ciy881 (PMC6603266; doi:10.1093/cid/ciy881)
Supplement: ciy881_suppl_Supplementary_Appendix [file ciy881_suppl_supplementary_appendix.docx]

**Supplementary literature review**

**Prevalence of pre-treatment drug resistance and impact on virological outcomes**

We searched PubMed for studies that reported on prevalence of either transmitted drug resistance (TDR) or pre-treatment drug resistance (PDR), as well as those studies that additionally reported on their impact on virological outcomes. The studies needed to have been published in Africa before 14 February 2017.

Search (((pretreatment) OR pre-treatment) OR transmitted) OR primary **(1,561,680)**

Search HIV **(313,095)**

Search Resistance **(711,925)**

Search Africa **(285,806)**

Search ((ART) OR Antiretroviral) OR anti-retroviral (**143,321**)

Combining all search criteria above using the Boolean operator ‘AND’ Search (((((((((pretreatment) OR pre-treatment) OR transmitted) OR primary)) AND HIV) AND Resistance) AND Africa)) AND (((ART) OR Antiretroviral) OR anti-retroviral)- **237**

We assessed the titles of all 237 articles and excluded 154 articles not related to the topic of interest. We read the abstract of the remaining 83 articles and selected 74 articles for full review. Of these 74 articles, a further 10 were excluded after reading the full articles. Two of the excluded studies were either reviews or meta-analysis[1, 2] , one was a mathematical model [3], one was a commentary on a published study [4], one evaluated resistance in children [5], one was on WHO early warning indicators [6], one was on acquired resistance [7], one was a multi-country sub-study of the START trial with no data on the African cohorts [8], one pooled sequence data from other published studies [9] and in one, the numerators were not very clear [10]. Of the remaining 64 published articles; 57 studies reported on prevalence of PDR only [11-67] whilst the remaining seven reported on both prevalence and impact of PDR on virological outcomes [68-74] (Supp. Table 1). These seven studies are heterogeneous and varied in size from 65 [72] to 2579 individuals [68, 70]. Six of the studies from the literature review identified drug resistance mutations using population sequencing [68-71, 73, 74] whilst two studies examined for the presence of minority drug resistant variants [69, 72].

The study by Boender et al [68] and Hamers et al [70] relate to the same cohort, used the same sample size and addressed outcomes which were only slightly different. In Hamers et al, the outcome was virological failure (VF) at 12 months whilst in the analysis by Boender et al, the outcome was time to treatment switch to second-line ART for presumed failure.

**Supp. Table 1: Published literature on the prevalence of pre-treatment drug resistance and the impact on virological outcomes**

| **Author** | **Study setting** | **Study population/Sample size** | **Study design** | **Year of sampling** | **Outcome** | **Prevalence/effect estimate** |
| --- | --- | --- | --- | --- | --- | --- |
| Boender, TS [68] | 13 clinical sites (PASER-M) in Kenya, Nigeria, South Africa, Uganda, Zambia & Zimbabwe | 2579 ART naïve HIV positive individuals initiating ART | Prospective cohort | 2007-2009 | Proportion with PDR  Impact of PDR on switching from 1^st^ to 2^nd^ line for presumed ART failure | 5.5% had PDR  PDR associated with switch to 2^nd^ line adjusted hazard ratio (aHR) 3.80 (95% CI 1.49-9.68) |
| Chung, M [69] | Nairobi, Kenya | 386 ART naïve individuals starting ART in a randomised trial of adherence. 356 initiated ART | Samples from a randomised trial  Fixed dose combination of D4T/3TC/NVP | 2006 | Proportion with PDR using Oligonucleotide ligation assay for point mutations of NNRTI ((K103N, Y181C and G190A) and 3TC (M184V) | 15/386 (3.9%) with PDR pre-ART.  13/356 (3.7%) with PDR in those who initiated ART. PDR associated with virological failure, Rate ratio 10.39 (95% CI, 3.23-32.41) |
| Hamers, R [70] | 13 clinical sites in Kenya, Nigeria,, South Africa, Uganda, Zambia, and Zimbabwe (PASER-M) | 2579 ART naïve HIV positive individuals initiating ART | Prospective cohort | March 2007 to September 2009 | Proportion with PDR;  Risk factors for virological failure (VL≥400 copies/mL) | 175/2579 (7%) with PDR;  213/2115 (10%) had virological failure at 12 months on ART:  PDR & fully active drug vs. no PDR; aOR 1.01 (95% CI 0.55-1.87);  PDR & partially active drug vs. no PDR aOR 2.13 (95% CI 1.44-3.14) |
| Lee, G [71] | Kampala and Mbarara, Uganda | 81 HIV positive ART naïve from Kampala; 491 from Mbarara | Prospective cohort D4T/3TC/NVP | Kampala: March 2002 to Dec 2004 Mbarara: 2005 to 2010 | Proportion with TDR | **Kampala**: 6/81 (7%) NRTI 3/81 (3.7%); NNRTI 1/81 (1.2%); PI 1/81 (1.2%)  **Mbarara:** 15/491 (3%) NRTI 4/491 (0.8%) NNRTI 9/491 (1.8% PI: None  Time to virological suppression (VL≤400 copies/mL) was similar in Kampala patients with and without PDR (97 days vs. 90 days; p=0.3) & for Mbarara patients PDR vs. No PDR (89 days vs. 85 days, p=0.05) |
| Mzingwane, M [72] | Pretoria, South Africa | 65 HIV-positive individuals who initiated ART and had at least one follow up viral load | Cohort study | July 2013 and May 2014 | Minority mutations on risk of virological failure (VL>1000 copies/mL after 6 months on ART) | 8/65 (12.3%) with VF. Deep sequencing to 1% in 4 individuals with failure, 3 with VS and 1 with low level viraemia. All 8 individuals harboured mutations compromising EFV. No difference in mutation frequency between the 4 with VF and 4 without VF. Active to both TDF/FTC |
| Rusine, J [73] | Kigali, Rwanda | 158 HIV positive individuals who initiated ART with 12 months VL available. 109 with baseline resistance test | Nested within a prospective study | November 2007 and January 2010 | Proportion with PDR.  Risk factors for virological failure at 12 months | 4/109 (3.6%)  No impact of PDR on risk of failure but very few had PDR |
| Kantor, R [74] | Brazil, Haiti, India, Malawi*, Peru, South Africa*, Thailand, USA, Zimbabwe* | Africa*-179 (38%), South America-93 (20%), Asia 88 (19%), USA 66 (14%), Caribbean 40 (9%) | Nested case-cohort study within a clinical trial | 2005-2007 | Proportion with baseline resistance Association between baseline drug resistance and VF (VL>1000 copies/mL) | 33/466 (7.1%)  Baseline drug resistance associated with VF; aHR 2.1 95% CI 1.0-4.6) |
| *Hong, S [75] | Namibia | 384 HIV-positive with baseline sequences. 239 on ART had PDR sequences at baseline and VL at 12 months | Prospective cohort | 2009 | Proportion with PDR  VF at 12 months | Any PDR 6.8%, NNRTI 6.3%, Both NRTI/NNRTI 0.3%, PI 0.3% Baseline NNRTI associated with VF; OR 8.8 (95% CI 2.6-29.9) |
| *Beck, I [76] | Kenya | 1,230 HIV-positive participants, 1227 with ARV data: 709 NVP-based ART, 518 EFV-based ART | Participants enrolled from three different studies | 2006, 2010, 2014 | PDR at enrolment 2% and 10% thresholds.  VF at 12 months | Any PDR 58/1,230 (4.7%) at 2% threshold; 47/1,230 (3.8%) at 10% threshold.  NVP-based ART vs. EFV-based ART: 2% threshold for presence of PDR; VF 27/38 (71.1%) vs. 5/20 (25%) at 2% threshold. 10% threshold for presence of PDR; VF 23/32 (71.9%) vs. 5/15 (33.3%) |
| *Zoufaly, A [77] | Cameroon | 300 patients started first-line ART. 238 had VL data at 12 months | Case control nested within a prospective study | Jan–Oct 2010 | VF at 12 months | 38/238 (16%) with VF at 12 months. 6/30 (20%) with VF and 6/35 (17%) matched controls without VF had PDR at 1% threshold using NGS (p=0.77) |

#We updated this literature review on 29 July 2018 and identified an additional 41 studies after combining all the search terms. None of these 41 studies examined the impact of pretreatment drug resistance (PDR) on virological outcomes. NRTI Nucleoside reverse transcriptase inhibitor; NNRTI Non-nucleoside reverse transcriptase inhibitor; TDF Tenofovir; FTC Emtricitabine; VL Viral load; VF Virological failure; OR Odds ratio; HR Hazard ratio; *studies identified outside the systematic review

In these studies, population-based sequencing of HIV protease and reverse transcriptase was done and drug resistance mutations defined using the International Antiviral Society USA mutation list of December, 2010. The study by Hamers and colleagues, reported that PDR to at least one prescribed drug vs. no PDR was associated with VF (VL ≥400 copies/mL) after 12 months on ART (OR 2.13, 95% CI 1.44–3.14). PDR on fully active drug compared to not having PDR was not associated with VF, OR (1.01, 95% CI: 0.55–1.87). The median number of active drugs to participants with PDR to at least one prescribed drug was 2 (IQR 1.25-2.0). A third of the participants in this study were on tenofovir (TDF) -containing regimen combined with either lamivudine (3TC) or emtricitabine (FTC) and the third agent was either nevirapine (NVP) or efavirenz (EFV). Nearly all the remaining individuals were either on a zidovudine (ZDV) or stavudine (D4T) backbone combined with either EFV or NVP.

In the other study by Chung et al [69] in Kenya, Oligonucleotide assay was used to test the pre-treatment samples for point mutations that confer resistance to NNRTI (K103N, Y181C, G190A) and 3TC (M184V) if present at the 2% detection threshold. PDR was present in 15 (3.9%) of 386 HIV-positive individuals assessed; 356 were initiated on a fixed dose combination D4T, 3TC and NVP within an adherence trial. They retrospectively examined the impact of transmitted drug resistance on virological failure (VL ≥1,000) in individuals who completed 18 months of follow up. Among the 356 individuals who started ART, 13 (3.7%) had PDR and 51 (14.3%) developed virological failure. Seven of 13 individuals with PDR developed VF whilst 44 of those without PDR developed VF. Two individuals amongst those with PDR had the M184V and at least one other NNRTI mutation. All the others had only NNRTI drug resistance mutation. In a multivariable cox regression model that adjusted for age, baseline viral load, adherence, employment and randomization assignment, PDR was independently associated with the risk of VF, HR 10.39 (95% CI, 3.23-32.41).

In the study by Rusine et al [73] in Kigali, Rwanda, 158 HIV-positive individuals who initiated ART as part of a larger prospective trial were evaluated for virological failure at 12 months. Population sequencing was done retrospectively on participants that had baseline and month 12 samples with a VL>1000 copies/mL. 89% of patients were on ZDV + 3TC with either NVP or EFV. 7% were on D4T and 4% on TDF containing regimen. 18 (11.4%) of 158 patients developed VF. Four patients had PDR; three of whom had only NNRTI mutations and one had both NNRTI and NRTI mutations. Of 91 individuals who achieved virological suppression and had pre-treatment genotype available, only 1 (1.1%) had PDR. Of 16 individuals with VF who had pre-treatment genotype available, 3 (18.8%) had PDR (p<0.001). However, in the univariable model, there was no association between PDR and VF. The number of people with PDR in this study was very small with potentially low power to detect a difference.

Lee et al [71], examined the impact of PDR on virological suppression (VL<400 copies/mL) at six months using retrospective samples from Kampala, an urban city, and Mbarara, a rural setting, in Uganda. Kaplan-Meier survival methods were used to estimate time to virological suppression in the groups with and without PDR. Population sequencing of the *pol* gene was carried out on baseline plasma samples.

Of the 74 Kampala patients (68 wildtype and 6 PDR) that were on ART (fixed dose combination of D4T, 3TC and NVP), 67/74 (91%) achieved suppression with a median time to suppression of 90 days (IQR 85-99). Of the 68 patients without PDR, 62 (91%) achieved suppression in a median of 90 days (IQR 84-99) whilst 5/6 (83%) with PDR achieved suppression in a median of 97 days (IQR 85-100) with no statistically significant difference in the group with and without PDR (log-rank test, p = 0.3). Of the six individuals with PDR, three had NNRTI, one NRTI, one PI and one had both NNRTI and NRTI PDR.

Of the 439 patients from Mbarara who were on ART and eligible, 13 had PDR, and 434 (99%) achieved virological suppression overall. Virological suppression was achieved in 422/426 (99%) patients without PDR and 12/13 (92%) patients with PDR at a median of 85 days (IQR 83–97) and 89 days (IQR 83–172), respectively (log rank test p= 0.05). Of the 13 individuals with PDR, eight had NNRTI, three NRTI and two had both NNRTI and NRTI PDR. 86% were on nevirapine based ART, and 12% on EFV in combination with 3TC and ZDV. The time to suppression estimates were not adjusted for baseline differences, but the authors commented that baseline characteristics did not differ significantly between the groups with and without PDR, except in the Kampala cohort in which the baseline CD4 count was significantly higher in those achieving virological suppression at 6 months than in those that did not (median 68 cells/mm^3^ vs. 19 cells/mm^3^, p=0.04).

The study by Mzingwane et al [72], with a sample size of 65 individuals on ART was equally too small to allow a firm conclusion to be drawn about the impact of PDR on virological outcomes. This is one of the very few studies in the African setting that has examined the impact of minority drug resistant variants on virological outcomes. Deep sequencing was done on pre-treatment samples from 8 HIV-positive individuals; four with virological failure (VL ≥1000copies/mL after 6 months on ART), three with virological suppression (VL < 50 copies/mL) and one with low level viraemia (VL<1000 copies/mL). All were on a fixed dose combination of TDF/FTC/EFV. The mutations identified at the 1% level of deep sequencing showed low to high level resistance to efavirenz. Although there were thymidine analogue mutations identified in all eight individuals as well as other NRTI mutations, the viral isolates were susceptible to TDF and FTC according to the Stanford algorithm. No conclusions can be drawn about the impact of minority variants on virological outcomes based on this small study.

The study by Kantor et al [74] was a multinational case cohort study nested within a clinical trial comprising 466 HIV-positive participants with genotype results; 38% of whom were from the Africa continent, mainly Southern Africa. PDR was ascertained using *pol* genotyping. The study examined the association between PDR and virological failure. At least one drug resistance mutation was detected in 24 (9.6%) of 256 participants with virological failure and 9 (4.3%) of 210 individuals without virological failure. Of the 24 with PDR amongst individuals with VF, 14 had NNRTI, nine both NNRTI and NRTI and one PI PDR. Of the nine with PDR amongst those without VF, one had NNRTI, seven had NNRTI and one PI PDR. In a multivariable model adjusting for sex, treatment arm, sex-treatment arm interaction, pretreatment CD4 cell count, baseline VL, subtype, CD4 cell count change and self-report of non-adherence within the first 12 weeks, PDR was significantly associated with VF and time to VF (HR, 1.4; 95% CI, .98–2.1). A third of patients were on EFV in combination with coformulated TDF and FTC, a third on didanosine, FTC and atazanavir and another third on EFV with coformulated ZDV and 3TC.

Three additional studies in the African setting were identified outside the systematic review from other sources[75-77]. The first study in Namibia [75] was amongst 394 HIV-positive individuals, of whom 384 had successful sequencing of their plasma samples at baseline using population sequencing; 6.8% had PDR to any drug, 6.3% to NNRTIs, 0.3% to both NNRTI and NRTI and 0.3% to PIs. Of the 394 individuals; 68% were on zidovudine and lamivudine combined with either EFV or NVP, 13% were on D4T and 3TC combined with either EFV or NVP, 11% were on TDF and 3TC combined with NVP, 6% were on ZDV, TDF, 3TC combined with EFV. The remaining were on protease inhibitor-based ART. Of the 384 individuals, 239 who were on first-line ART had PDR data at baseline and viral load at 12 months. Baseline NNRTI PDR was associated with virological failure at 12 months on univariable analysis [OR 8.8 (95% CI 2.6, 29.9)]. No multivariable analysis was undertaken.

The second study was conference poster examining the relationship between PDR and virological failure at 12 months in HIV-positive individuals on EFV or NVP-based ART in Kenya [76]. The NRTI backbone was not specified. Oligonucleotide assay was used to determine the presence of NNRTI (K103N Y181C G190A), NRTI M184V (3TC) and K65R (TDF) PDR at a threshold of ≥2%.

There were 709 HIV-positive individuals on NVP-based ART. Thirty-eight (5.3%) had any PDR at the 2% threshold; 23 had only NNRTI and 15 both NNRTI and NNRTI PDR. 13.7% experienced virological failure.

There were 509 HIV-positive individuals in EFV-based ART. Twenty (3.9%) had any PDR at the 2% threshold; 13 had only NNRTI and 6 had both NNRTI and NRTI PDR.

PDR frequency of ≥10% within a subject quasi-species was associated with VF at 12 months in those with NVP and EFV-based ART whilst a PDR frequency of 2-9% was associated with VF in those with NVP-based but not EFV-based ART. Furthermore, the presence of NNRTI PDR only was associated with VF in those on NVP-based ART but not EFV-based ART which have VF rates similar to those with wild type virus. Presence of both NNRTI and NRTI PDR was associated with VF in both EFV and NVP-Based ART. Although this was a descriptive study with no adjustment for confounders, the authors concluded that the lower rate of VF amongst those receiving EFV-based ART, regardless of the presence or absence of PDR indicates the greater potency of EFV-based ART compared to NVP-based ART.

The study by Zoufaly et al [77] was a case-control study nested within a prospective cohort to examine the risk factors for virological failure (VL ≥ 1000 copies/mL) at 12 months in 300 individuals consecutively started on ART between January and October 2010 in rural Cameroon. Of the 238 individuals who had been on ART for 12 months, 38 (16%) experienced virological failure. Pre-treatment (baseline) resistance using next generation sequencing with a detection threshold of 1% was assessed in patients with virological failure and compared with an equal number of controls that had VL < 1000 copies/mL matched for age, sex, CD4 count and viral load at baseline. 18% of patients were on a TDF containing ART combined with either 3TC or FTC. The majority were on ZDV/3TC (81%) containing regimen. The third agent was NVP in 71% of cases, EFV (25%) and boosted lopinavir (4.2%). At baseline, 6/30 (20%) patients with VF and 6/35 (17%) of controls had evidence of minority drug resistance mutations detected at 1% threshold using NGS (p=0.77). In multivariable regression, the independent risk factors for VF were a lower baseline CD4 cell count (OR 1.47, 95% CI 1.02–2.08, per 100 cells/mm^3^ lower) and a lower pill count adherence (OR 1.04, 95% CI 1.02–1.07 per 1% lower).

This literature review has a few limitations. We restricted our search to PubMed and included only studies published in English. Hence we could have missed relevant studies in other databases or published in another language.

To summarize, four analytical studies (two from the same cohort) in the African setting [68-70, 74] showed an association between the presence of PDR and virological failure. PDR was defined as any resistance mutation that compromised the activity of one or more of the ART components, with the presence of only NNRTI PDR being sufficient to result in VF. These studies were mainly those with larger sample sizes. None of the participants in these studies were on a single tablet regiment of TDF, FTC and EFV and in the two studies in which TDF and FTC were part of the ART combination, EFV or NVP was the third agent [70, 74]. The majority of participants were on either ZDV or d4T backbone combined with either EFV or NVP. Two descriptive studies showed an association between PDR and VF [75, 76] with one of them suggesting that both NRTI and NNRTI PDR need to be present to compromise EFV-based ART [76]. Four of these studies accounted for adherence to ART [68-70, 74]**.**

Four small sample-sized studies did not show an association between PDR and virological outcome [71-73, 77]. Two of these studies identified drug resistance mutations using next generation sequencing [72] [77].

We present findings from the largest nested prospective study in the African continent examining the association between PDR, identified by next generation sequencing, and virological outcomes in HIV-positive individuals primarily on the WHO recommended first-line ART comprising a fixed dose combination of tenofovir, emtricitabine and efavirenz.

**Supplementary Methods**

1. **Illumina sequencing**

Ribonucleic acids were extracted from 1ml of plasma with a VL >1000 copies/ml, using the QIAamp Viral RNA mini kit (Qiagen, Hilden, Germany), and were eluted in 60μl of elution buffer. The near full HIV genome was amplified with 4 subtype C specific primers pairs (Supp. Table 2), generating 4 overlapping amplicons of 2,1, 2,3, 2,2 and 3,9kb, with an adapted protocol previously described by Gall et al [78].

DNA concentrations of amplicons were quantified with the Qubit dsDNA HS Assay kit (Invitrogen, Carlsbad, CA). Diluted amplicons were pooled equimolarly and prepared for library using the Nextera XT DNA Library preparation and the Nextera XT DNA sample preparation index kits (Illumina, San Diego, CA), following the manufacturer’s protocol. The runs comprised a total of 96 samples, including 3 controls: one negative, one inter-run and one intra-run samples. If amplification or sequencing failed for the *pol* region of the HIV genome, samples were re-amplified with only the PANA2 primers and sequenced in a 386 samples’ run, with the same controls as the 96 run.

1. **Validation of next generation sequencing data**

Quality controls of the fastq files were done with the FASTQC [79] and QUASR [80] software. Reads assemblies were performed on Geneious 10.0.6 software: reads between 100 and 300bps were selected and excluded if Phred score <30, then trimmed up to 10bp from 5’ and 30bp at the 3’ end, and finally were mapped against a subtype C reference sequence (AF411967) annotated for WHO Surveillance of DRM.

**Supp. Table 2: Primers used for near full-length amplification of HIV genome**

| **Primer** | **Sequence (5'-3')** | **Position (nt)*** | **Product size (bp)*** |
| --- | --- | --- | --- |
| PAN1_Fwd | AGCCYGGGAGCTCTCTG | 483-496 | 2144 |
| PANC1_Rev | TGTCAATGGCCATTGTTTAACCTTTGG | 2604-2627 |  |
| PANA2_Fwd | GAGGCAATGAGCCAARCAAACA | 1885-1903 | 2277 |
| PANA2_Rev | TGTGCTGGTACCCATGACA | 4147-4162 |  |
| PANA3_Fwd | AGCATAGTAATATGGGGAAAGACTC | 3687-3708 | 2175 |
| PANA3_Rev | TTCCAGGGCTCTAGKTTAGG | 5846-5862 |  |
| PANA4_Fwd | AASCCCCAGAGGAYCAGG | 5569-5583 | 3948 |
| PANC4_Rev | CTTATATGCAGCATCTGAGGG | 9497-9517 |  |

* according to HIV-1 reference strain HXB2. nt=nucleotide, bp=base pair

The intra-run and inter-run controls were used to assess the reproducibility and accuracy of our method (n=29). The mean identity of the consensus sequences derived for each duplicate was 99.81% (standard deviation [SD]=0.35%). The mean difference between the single nucleotide polymorphisms frequencies, detected from 0.2% to 100%, was 1.4% (SD=2.5%); therefore, we estimated PDR prevalence by accounting for DRM detected at the thresholds of 5% (according to our accuracy analysis) and 20% (detection limit of gold standard Sanger sequencing), with a minimum of 20 reads comprising the DRM.

**Supplementary Results**

1. **Description of drug resistance mutations detected at 20% detection threshold**

Amongst the 109/1,148 (9.5%) individuals with any PDR detected by NGS at 20% level, there were 138 DRM; of which 118/138 (85.5%) belonged to the NNRTI class, mostly represented by the K103N/S mutation (n=80/118, 67.8%), while 12/138 (8.7%) and 8/138 (5.8%) were in the NRTI and PI classes respectively (Supplementary Figure 1). The main mutations found in the NRTI class were M184V (n=5/12, 41.7%) associated with lamivudine/emtricitabine resistance, and tenofovir associated resistance mutation K65R (n=2/12, 16.7%). They were followed by the thymidine analog mutation (TAM) T215S (2/12, 16.7%) and D67N (n=1/13, 8%). Non-polymorphic PI resistance mutations were also detected. With the exception of the D30N mutation (2/8, 25%), selected by the PI nelfinavir that was used in South Africa few years ago for 2^nd^-line therapy, it is unlikely these PI mutations were selected or transmitted, due to the limited use of PI regimen in our setting. Therefore, they could represent either some amplification errors or the dynamics of HIV-1 genetic diversity.

**Supplementary Figure 1** Distribution of DRM (n=138) detected at 20% level among all ART naïve participants harboring resistance mutations detected by NGS. In the inner circle figured the distribution of the ARV classes, and in the outer circle figured the DRM.

1. **Description of drug resistance mutations detected at 5% detection threshold**

Amongst the 147/1,148 (12.8%) individuals with any PDR detected by NGS at 5% level, there were 199 DRM.

Resistance mutations to NNRTI represented 79.4% (158/199) of all DRM, and NRTI and PI resistance mutations represented respectively 12.6% (25/199) and 8.0% (16/199) of all DRM (Supplementary Figure 2). In the NRTI class, the TAM D67GN (7/25, 28%) was in the majority followed by M184IV mutation (6/25, 24%) and K65R (5/25, 20%). Non-polymorphic PI resistance mutations were more detected, with a majority of M46IL (4/16, 25%).

**Supplementary Figure 2** Distribution of DRM (n=199) detected at 5% level among all ART naïve participants harboring resistance mutations detected by NGS. In the inner circle figured the distribution of the ARV classes, and in the outer circle figured the DRM.

1. **Dual class resistance NRTI/NNRTI**

A total of 6 and 11 of 1148 participants displayed dual resistance to NRTI and NNRTI at the 20% and 5% detection thresholds which corresponds to 6/109 (5.5%) and 11/147 (7.8%) amongst those harboring drug resistance mutations respectively (Supplementary Table 3).

**Supp. Table 3: Participants harboring dual class NRTI/NNRTI drug resistance mutations**

| **ID** | **NRTI** | | **NNRTI** | | |
| --- | --- | --- | --- | --- | --- |
| 1 | **K65R** | **V75M** | **K103N/S** | **V106M** | **Y181C** |
| 2 | **D67N** | **M184V** | **V106M** | **Y188C** |  |
| 3 | **K65R** | **M1814V** | **Y181C** | **Y188L** |  |
| 4 | **L74I** | **M184V** | **K103N** | **P225H** |  |
| 5 | **M184V** |  | **K103N** | **P225H** |  |
| 6 | **M184V** |  | **K103N** | **P225H** |  |
| 7 | *K65R* |  | **K101E** | **Y188C** |  |
| 8 | *K65R* | *K219E* | **K103N** | **Y181C** |  |
| 9 | *D67N* |  | **K103N/S** | **V106M** |  |
| 10 | *K65R* |  | **K103N** | *V106M* |  |
| 11 | *K70R* |  | **K103N** |  |  |

Mutations detected >20% are represented in bold. Mutations detected between 5 and 20% are represented in italic.

1. **Number of individuals with VL results at each visit, overall and by PDR status**

Viral load results were fairly complete up to 6m (Supp. Table 4). At 12m, a slightly higher proportion of individuals with no PDR had a VL result than those with PDR, but the difference was not statistically significant. After 12 months, over half of the participants did not have viral load results as a result of later enrolment in the cohort so were censored at their last measured viral load.

**Supp. Table 4: Number of individuals with VL results at each visit, overall and by PDR status**

| **Month** | **n with VL (%) - All (N=837)** | **n with VL (%) - any PDR (N=162)** | **n with VL (%) - no PDR (N=675)** |
| --- | --- | --- | --- |
| Pre-ART | 829 (99.0%) | 161 (99.4%) | 668 (99.0%) |
| 2m | 588 (70.3%) | 114 (70.4%) | 474 (70.2%) |
| 6m | 713 (85.2%) | 139 (85.8%) | 574 (85.0%) |
| 12m | 553 (66.1%) | 99 (61.1%) | 454 (67.3%) |
| 18m | 331 (39.5%) | 60 (37.0%) | 271 (40.1%) |
| 24+ m | 403 (48.1%) | 57 (35.2%) | 346 (51.3%) |

Individuals with no PDR were more likely to have a VL result after 12 months but this is unlikely to have affected our findings as 75% of the participants were suppressed by 5.8 months.

**References**

1. Ssemwanga D, Lihana RW, Ugoji C, et al. Update on HIV-1 acquired and transmitted drug resistance in Africa. AIDS Rev **2015**; 17(1): 3-20.

2. Rhee SY, Blanco JL, Jordan MR, et al. Geographic and temporal trends in the molecular epidemiology and genetic mechanisms of transmitted HIV-1 drug resistance: an individual-patient- and sequence-level meta-analysis. PLoS Med **2015**; 12(4): e1001810.

3. Wilson DP, Kahn J, Blower SM. Predicting the epidemiological impact of antiretroviral allocation strategies in KwaZulu-Natal: the effect of the urban-rural divide. Proc Natl Acad Sci U S A **2006**; 103(38): 14228-33.

4. Parikh UM, Mellors JW. Pretreatment HIV-1 drug resistance is strongly associated with virologic failure in HIV-infected patients receiving partly active antiretroviral regimens. Future Microbiol **2012**; 7(8): 929-32.

5. Ceccarelli L, Salpini R, Moudourou S, et al. Characterization of drug resistance mutations in naive and ART-treated patients infected with HIV-1 in Yaounde, Cameroon. J Med Virol **2012**; 84(5): 721-7.

6. Fokam J, Elat JB, Billong SC, et al. Monitoring HIV Drug Resistance Early Warning Indicators in Cameroon: A Study Following the Revised World Health Organization Recommendations. PLoS One **2015**; 10(6): e0129210.

7. Hanson DL, Adje-Toure C, Talla-Nzussouo N, et al. HIV type 1 drug resistance in adults receiving highly active antiretroviral therapy in Abidjan, Cote d'Ivoire. AIDS Res Hum Retroviruses **2009**; 25(5): 489-95.

8. Baxter JD, Dunn D, White E, et al. Global HIV-1 transmitted drug resistance in the INSIGHT Strategic Timing of AntiRetroviral Treatment (START) trial. HIV Med **2015**; 16 Suppl 1: 77-87.

9. Manasa J, Katzenstein D, Cassol S, et al. Primary drug resistance in South Africa: data from 10 years of surveys. AIDS Res Hum Retroviruses **2012**; 28(6): 558-65.

10. Msimanga PW, Vardas E, Engelbrecht S. HIV-1 diversity in an antiretroviral treatment naive cohort from Bushbuckridge, Mpumalanga Province, South Africa. Virol J **2015**; 12: 24.

11. Elmi Abar A, Jlizi A, Darar HY, Kacem MA, Slim A. HIV-1 drug resistance genotyping from antiretroviral therapy (ART) naive and first-line treatment failures in Djiboutian patients. Diagn Pathol **2012**; 7: 138.

12. Abegaz WE, Grossman Z, Wolday D, et al. Threshold survey evaluating transmitted HIV drug resistance among public antenatal clinic clients in Addis Ababa, Ethiopia. Antivir Ther **2008**; 13 Suppl 2: 89-94.

13. Bila DC, Young P, Merks H, et al. Evolution of primary HIV drug resistance in a subtype C dominated epidemic in Mozambique. PLoS One **2013**; 8(7): e68213.

14. Afonso JM, Bello G, Guimaraes ML, Sojka M, Morgado MG. HIV-1 genetic diversity and transmitted drug resistance mutations among patients from the North, Central and South regions of Angola. PLoS One **2012**; 7(8): e42996.

15. Aghokeng AF, Kouanfack C, Laurent C, et al. Scale-up of antiretroviral treatment in sub-Saharan Africa is accompanied by increasing HIV-1 drug resistance mutations in drug-naive patients. AIDS **2011**; 25(17): 2183-8.

16. Aghokeng AF, Vergne L, Mpoudi-Ngole E, et al. Evaluation of transmitted HIV drug resistance among recently-infected antenatal clinic attendees in four Central African countries. Antivir Ther **2009**; 14(3): 401-11.

17. Ajoge HO, Gordon ML, Ibrahim S, Shittu OS, Ndung'u T, Olonitola SO. Drug resistance pattern of HIV type 1 isolates sampled in 2007 from therapy-naive pregnant women in North-Central Nigeria. AIDS Res Hum Retroviruses **2012**; 28(1): 115-8.

18. Ayouba A, Lien TT, Nouhin J, et al. Low prevalence of HIV type 1 drug resistance mutations in untreated, recently infected patients from Burkina Faso, Cote d'Ivoire, Senegal, Thailand, and Vietnam: the ANRS 12134 study. AIDS Res Hum Retroviruses **2009**; 25(11): 1193-6.

19. Bartolo I, Zakovic S, Martin F, et al. HIV-1 diversity, transmission dynamics and primary drug resistance in Angola. PLoS One **2014**; 9(12): e113626.

20. Bartolo I, Casanovas J, Bastos R, et al. HIV-1 genetic diversity and transmitted drug resistance in health care settings in Maputo, Mozambique. J Acquir Immune Defic Syndr **2009**; 51(3): 323-31.

21. Bonney EY, Addo NA, Ntim NA, et al. Low level of transmitted HIV Drug resistance at two HIV care centres in Ghana: a threshold survey. Ghana Med J **2013**; 47(2): 82-6.

22. Bruzzone B, Saladini F, Sticchi L, et al. Prevalence of HIV-1 Subtypes and Drug Resistance-Associated Mutations in HIV-1-Positive Treatment-Naive Pregnant Women in Pointe Noire, Republic of the Congo (Kento-Mwana Project). AIDS Res Hum Retroviruses **2015**; 31(8): 837-40.

23. Burda ST, Viswanath R, Zhao J, et al. HIV-1 reverse transcriptase drug-resistance mutations in chronically infected individuals receiving or naive to HAART in Cameroon. J Med Virol **2010**; 82(2): 187-96.

24. Bussmann H, de la Hoz Gomez F, Roels TH, et al. Prevalence of transmitted HIV drug resistance in Botswana: lessons learned from the HIVDR-Threshold Survey conducted among women presenting for routine antenatal care as part of the 2007 national sentinel survey. AIDS Res Hum Retroviruses **2011**; 27(4): 365-72.

25. Castelbranco EP, da Silva Souza E, Cavalcanti AM, Martins AN, de Alencar LC, Tanuri A. Frequency of primary resistance to antiretroviral drugs and genetic variability of HIV-1 among infected pregnant women recently diagnosed in Luanda-Angola. AIDS Res Hum Retroviruses **2010**; 26(12): 1313-6.

26. Charpentier C, Bellecave P, Cisse M, et al. High prevalence of antiretroviral drug resistance among HIV-1-untreated patients in Guinea-Conakry and in Niger. Antivir Ther **2011**; 16(3): 429-33.

27. Derache A, Maiga AI, Traore O, et al. Evolution of genetic diversity and drug resistance mutations in HIV-1 among untreated patients from Mali between 2005 and 2006. J Antimicrob Chemother **2008**; 62(3): 456-63.

28. Derache A, Traore O, Koita V, et al. Genetic diversity and drug resistance mutations in HIV type 1 from untreated patients in Bamako, Mali. Antivir Ther **2007**; 12(1): 123-9.

29. Eshleman SH, Laeyendecker O, Parkin N, et al. Antiretroviral drug susceptibility among drug-naive adults with recent HIV infection in Rakai, Uganda. AIDS **2009**; 23(7): 845-52.

30. Fokam J, Takou D, Santoro MM, et al. Short Communication: Population-Based Surveillance of HIV-1 Drug Resistance in Cameroonian Adults Initiating Antiretroviral Therapy According to the World Health Organization Guidelines. AIDS Res Hum Retroviruses **2016**; 32(4): 329-33.

31. Haidara A, Chamberland A, Sylla M, et al. High level of primary drug resistance in Mali. HIV Med **2010**; 11(6): 404-11.

32. Hamers RL, Wallis CL, Kityo C, et al. HIV-1 drug resistance in antiretroviral-naive individuals in sub-Saharan Africa after rollout of antiretroviral therapy: a multicentre observational study. Lancet Infect Dis **2011**; 11(10): 750-9.

33. Handema R, Terunuma H, Kasolo F, et al. Prevalence of drug-resistance-associated mutations in antiretroviral drug-naive Zambians infected with subtype C HIV-1. AIDS Res Hum Retroviruses **2003**; 19(2): 151-60.

34. Hassan AS, Mwaringa SM, Obonyo CA, et al. Low prevalence of transmitted HIV type 1 drug resistance among antiretroviral-naive adults in a rural HIV clinic in Kenya. AIDS Res Hum Retroviruses **2013**; 29(1): 129-35.

35. Huang KH, Goedhals D, Fryer H, et al. Prevalence of HIV type-1 drug-associated mutations in pre-therapy patients in the Free State, South Africa. Antivir Ther **2009**; 14(7): 975-84.

36. Hunt GM, Ledwaba J, Basson AE, et al. Surveillance of transmitted HIV-1 drug resistance in Gauteng and KwaZulu-Natal Provinces, South Africa, 2005-2009. Clin Infect Dis **2012**; 54 Suppl 4: S334-8.

37. Huruy K, Maier M, Mulu A, Liebert UG. Limited increase in primary HIV-1C drug resistance mutations in treatment naive individuals in Ethiopia. J Med Virol **2015**; 87(6): 978-84.

38. Kamoto K, Aberle-Grasse J. Surveillance of transmitted HIV drug resistance with the World Health Organization threshold survey method in Lilongwe, Malawi. Antivir Ther **2008**; 13 Suppl 2: 83-7.

39. Kantor R, DeLong A, Balamane M, et al. HIV diversity and drug resistance from plasma and non-plasma analytes in a large treatment programme in western Kenya. J Int AIDS Soc **2014**; 17: 19262.

40. Kasang C, Kalluvya S, Majinge C, et al. HIV drug resistance (HIVDR) in antiretroviral therapy-naive patients in Tanzania not eligible for WHO threshold HIVDR survey is dramatically high. PLoS One **2011**; 6(8): e23091.

41. Koizumi Y, Ndembi N, Miyashita M, et al. Emergence of antiretroviral therapy resistance-associated primary mutations among drug-naive HIV-1-infected individuals in rural western Cameroon. J Acquir Immune Defic Syndr **2006**; 43(1): 15-22.

42. Maiga AI, Fofana DB, Maiga AC, et al. Transmitted antiretroviral drug resistance in newly HIV-infected and untreated patients in Segou and Bamako, Mali. AIDS Res Hum Retroviruses **2013**; 29(1): 182-6.

43. Manasa J, Danaviah S, Lessells R, et al. Increasing HIV-1 Drug Resistance Between 2010 and 2012 in Adults Participating in Population-Based HIV Surveillance in Rural KwaZulu-Natal, South Africa. AIDS Res Hum Retroviruses **2016**; 32(8): 763-9.

44. Maphalala G, Okello V, Mndzebele S, et al. Surveillance of transmitted HIV drug resistance in the Manzini-Mbabane corridor, Swaziland, in 2006. Antivir Ther **2008**; 13 Suppl 2: 95-100.

45. Masimba P, Kituma E, Klimkait T, et al. Prevalence of drug resistance mutations and HIV type 1 subtypes in an HIV type 1-infected cohort in rural Tanzania. AIDS Res Hum Retroviruses **2013**; 29(9): 1229-36.

46. Mensch BS, Gorbach PM, Kelly C, et al. Characteristics Associated with HIV Drug Resistance Among Women Screening for an HIV Prevention Trial in KwaZulu-Natal, South Africa. AIDS Behav **2015**; 19(11): 2076-86.

47. Mungati M, Mhangara M, Gonese E, et al. Pre-treatment drug resistance among patients initiating antiretroviral therapy (ART) in Zimbabwe: 2008-2010. BMC Res Notes **2016**; 9: 302.

48. Nazziwa J, Njai HF, Ndembi N, et al. Short communication: HIV type 1 transmitted drug resistance and evidence of transmission clusters among recently infected antiretroviral-naive individuals from Ugandan fishing communities of Lake Victoria. AIDS Res Hum Retroviruses **2013**; 29(5): 788-95.

49. Ndembi N, Hamers RL, Sigaloff KC, et al. Transmitted antiretroviral drug resistance among newly HIV-1 diagnosed young individuals in Kampala. AIDS **2011**; 25(7): 905-10.

50. Ndembi N, Lyagoba F, Nanteza B, et al. Transmitted antiretroviral drug resistance surveillance among newly HIV type 1-diagnosed women attending an antenatal clinic in Entebbe, Uganda. AIDS Res Hum Retroviruses **2008**; 24(6): 889-95.

51. Nwobegahay J, Selabe G, Ndjeka NO, Manhaeve C, Bessong PO. Low prevalence of transmitted genetic drug resistance in a cohort of HIV infected naive patients entering antiretroviral treatment programs at two sites in northern South Africa. J Med Virol **2012**; 84(12): 1839-43.

52. Nyombi BM, Holm-Hansen C, Kristiansen KI, Bjune G, Muller F. Prevalence of reverse transcriptase and protease mutations associated with antiretroviral drug resistance among drug-naive HIV-1 infected pregnant women in Kagera and Kilimanjaro regions, Tanzania. AIDS Res Ther **2008**; 5: 13.

53. Onsongo S, Abidi SH, Khamadi S, et al. Prevalence of Transmitted Drug Resistance Mutations in HIV-1-Infected Drug-Naive Patients from Urban and Suburban Regions of Kenya. AIDS Res Hum Retroviruses **2016**; 32(3): 220-5.

54. Onywera H, Maman D, Inzaule S, et al. Surveillance of HIV-1 pol transmitted drug resistance in acutely and recently infected antiretroviral drug-naive persons in rural western Kenya. PLoS One **2017**; 12(2): e0171124.

55. Parboosing R, Naidoo A, Gordon M, Taylor M, Vella V. Resistance to antiretroviral drugs in newly diagnosed, young treatment-naive HIV-positive pregnant women in the province of KwaZulu-Natal, South Africa. J Med Virol **2011**; 83(9): 1508-13.

56. Pillay V, Ledwaba J, Hunt G, et al. Antiretroviral drug resistance surveillance among drug-naive HIV-1-infected individuals in Gauteng Province, South Africa in 2002 and 2004. Antivir Ther **2008**; 13 Suppl 2: 101-7.

57. Rowley CF, MacLeod IJ, Maruapula D, et al. Sharp increase in rates of HIV transmitted drug resistance at antenatal clinics in Botswana demonstrates the need for routine surveillance. J Antimicrob Chemother **2016**; 71(5): 1361-6.

58. Sigaloff KC, Mandaliya K, Hamers RL, et al. Short communication: High prevalence of transmitted antiretroviral drug resistance among newly HIV type 1 diagnosed adults in Mombasa, Kenya. AIDS Res Hum Retroviruses **2012**; 28(9): 1033-7.

59. Somda A, Sangare L, Soro M, et al. Surveillance of transmitted drug-resistant HIV among young pregnant women in Ouagadougou, Burkina Faso. Clin Infect Dis **2012**; 54 Suppl 4: S317-9.

60. Somi GR, Kibuka T, Diallo K, et al. Surveillance of transmitted HIV drug resistance among women attending antenatal clinics in Dar es Salaam, Tanzania. Antivir Ther **2008**; 13 Suppl 2: 77-82.

61. Ssemwanga D, Kapaata A, Lyagoba F, et al. Low drug resistance levels among drug-naive individuals with recent HIV type 1 infection in a rural clinical cohort in southwestern Uganda. AIDS Res Hum Retroviruses **2012**; 28(12): 1784-7.

62. Steegen K, Carmona S, Bronze M, et al. Moderate Levels of Pre-Treatment HIV-1 Antiretroviral Drug Resistance Detected in the First South African National Survey. PLoS One **2016**; 11(12): e0166305.

63. Tebit DM, Sangare L, Tiba F, et al. Analysis of the diversity of the HIV-1 pol gene and drug resistance associated changes among drug-naive patients in Burkina Faso. J Med Virol **2009**; 81(10): 1691-701.

64. Tshabalala M, Manasa J, Zijenah LS, et al. Surveillance of transmitted antiretroviral drug resistance among HIV-1 infected women attending antenatal clinics in Chitungwiza, Zimbabwe. PLoS One **2011**; 6(6): e21241.

65. Wadonda-Kabondo N, Banda R, Moyo K, et al. Prevalence of transmitted HIV drug resistance among newly diagnosed antiretroviral therapy-naive pregnant women in Lilongwe and Blantyre, Malawi. Clin Infect Dis **2012**; 54 Suppl 4: S324-7.

66. Weidle PJ, Kityo CM, Mugyenyi P, et al. Resistance to antiretroviral therapy among patients in Uganda. J Acquir Immune Defic Syndr **2001**; 26(5): 495-500.

67. Bussmann H, Novitsky V, Wester W, et al. HIV-1 subtype C drug-resistance background among ARV-naive adults in Botswana. Antivir Chem Chemother **2005**; 16(2): 103-15.

68. Boender TS, Hoenderboom BM, Sigaloff KC, et al. Pretreatment HIV drug resistance increases regimen switches in sub-Saharan Africa. Clin Infect Dis **2015**; 61(11): 1749-58.

69. Chung MH, Beck IA, Dross S, et al. Oligonucleotide ligation assay detects HIV drug resistance associated with virologic failure among antiretroviral-naive adults in Kenya. J Acquir Immune Defic Syndr **2014**; 67(3): 246-53.

70. Hamers RL, Schuurman R, Sigaloff KC, et al. Effect of pretreatment HIV-1 drug resistance on immunological, virological, and drug-resistance outcomes of first-line antiretroviral treatment in sub-Saharan Africa: a multicentre cohort study. Lancet Infect Dis **2012**; 12(4): 307-17.

71. Lee GQ, Bangsberg DR, Muzoora C, et al. Prevalence and virologic consequences of transmitted HIV-1 drug resistance in Uganda. AIDS Res Hum Retroviruses **2014**; 30(9): 896-906.

72. Mzingwane ML, Tiemessen CT, Richter KL, Mayaphi SH, Hunt G, Bowyer SM. Pre-treatment minority HIV-1 drug resistance mutations and long term virological outcomes: is prediction possible? Virol J **2016**; 13(1): 170.

73. Rusine J, Asiimwe-Kateera B, van de Wijgert J, et al. Low primary and secondary HIV drug-resistance after 12 months of antiretroviral therapy in human immune-deficiency virus type 1 (HIV-1)-infected individuals from Kigali, Rwanda. PLoS One **2013**; 8(8): e64345.

74. Kantor R, Smeaton L, Vardhanabhuti S, et al. Pretreatment HIV Drug Resistance and HIV-1 Subtype C Are Independently Associated With Virologic Failure: Results From the Multinational PEARLS (ACTG A5175) Clinical Trial. Clin Infect Dis **2015**; 60(10): 1541-9.

75. Hong SY, Jonas A, DeKlerk M, et al. Population-based surveillance of HIV drug resistance emerging on treatment and associated factors at sentinel antiretroviral therapy sites in Namibia. J Acquir Immune Defic Syndr **2015**; 68(4): 463-71.

76. Beck I, Levine M, Milne R, et al. Impact of Pre-Treatment HIV-Drug Resistance on Virologic Outcome of First-Line NNRTI-ART. CROI 2017. Seattle, USA.

77. Zoufaly A, Jochum J, Hammerl R, et al. Virological failure after 1 year of first-line ART is not associated with HIV minority drug resistance in rural Cameroon. J Antimicrob Chemother **2015**; 70(3): 922-5.

78. Gall A, Ferns B, Morris C, et al. Universal amplification, next-generation sequencing, and assembly of HIV-1 genomes. J Clin Microbiol **2012**; 50(12): 3838-44.

79. S A. FastQC: a quality control tool for high throughput sequence data. Available at: <http://www.bioinformatics.babraham.ac.uk/projects/fastqc>.

80. Gaidatzis D, Lerch A, Hahne F, Stadler MB. QuasR: quantification and annotation of short reads in R. Bioinformatics **2015**; 31(7): 1130-2.
